# Upcycling Bread Waste into a Ag-Doped Carbon Material Applied to the Detection of Halogenated Compounds in Waters

**DOI:** 10.1021/acsami.2c08332

**Published:** 2022-08-23

**Authors:** Wenchao Duan, César Fernández-Sánchez, Martí Gich

**Affiliations:** † Institut de Ciència de Materials de Barcelona, ICMAB (CSIC), Campus UAB, 08193 Bellaterra, Spain; ‡ Institut de Microelectrònica de Barcelona, IMB-CNM (CSIC), Campus UAB, 08193 Bellaterra, Spain; § CIBER de Bioingeniería, Biomateriales y Nanomedicina (CIBER-BBN), Jordi Girona 18-26, 08034 Barcelona, Spain

**Keywords:** bread waste, silver nanoparticles, carbon electrode, electrochemical sensor, halides, sucralose, trichloroacetic acid

## Abstract

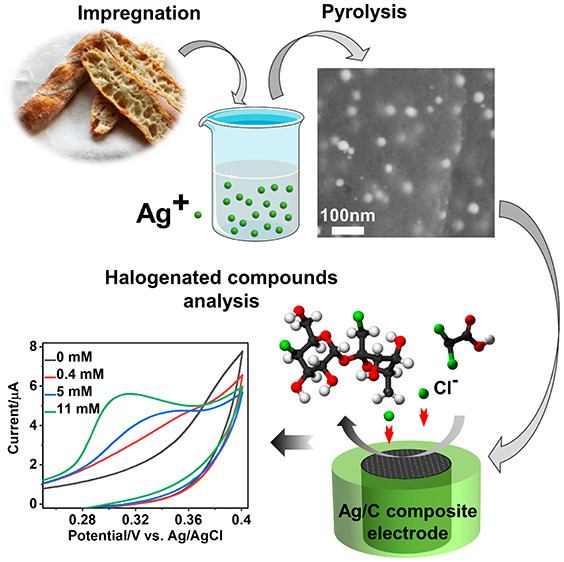

Bread waste is a major part of food wastage which could be upcycled to produce functional materials, following the principles of the circular bioeconomy. This work shows that bread waste can be recycled and valorized to produce a composite conductive material with excellent properties for chemical sensor applications. Here, dry bread is impregnated with an aqueous solution of a silver precursor and pyrolyzed to produce a porous carbon matrix containing Ag nanoparticles with diameters ranging from 20 to 40 nm. These particles perform as catalytic redox centers for the electrochemical detection of halide ions (Cl^–^, Br^–^, and I^–^) and organohalide target molecules such as sucralose and trichloroacetic acid. A thorough analytical characterization is carried out to show the potential application of the developed material for the manufacturing of electrochemical sensor approaches. The material preparation is sustainable, low-cost, simple, and upscalable. These are ideal features for the large-scale manufacturing by screen-printing technologies of single-use electrochemical sensors for the rapid analysis of halogenated organic pollutants in waters.

## Introduction

The increasing awareness of the environmental global challenges prompted the development of different policies to address them. For instance, the European Green deal roadmap, a political commitment for the EU to become climate-neutral by 2050, has recently been transformed into binding obligations through the EU Climate Law endorsed by the Parliament.^1^ The circular use of biomass is among the measures for its implementation and aims at promoting resource efficiency and stimulating the production of high added-value products from waste. In this context, one can anticipate the implementation of new initiatives enforcing the use of biowaste feedstocks in manufacturing processes.

Bread goes stale rapidly and thus constitutes one of the major fractions of food waste.^2^ Despite the progress that might be achieved in reducing its wastage, the bread will certainly remain a relevant, widely available bioresource with great upcycling potential. Since bread contains up to 10 wt % protein and 70 wt % carbohydrates, in particular starch,^3^ one of the better explored upcycling alternatives has been the fermentative production of value-added products^2^ such as chemicals,^4^ fuels^5^ and enzymes.^6^ Interestingly, it has been shown that starch can be used for the production of porous carbon materials.^7−9^ This opens new opportunities for the valorization of bread waste into carbon materials that are commonly prepared from toxic and expensive chemicals such as resorcinol, polypyrrole or p-nitrophenol.^10^ An additional advantage of the direct carbonization of bioresources is that it can provide a sustainable and simple method to prepare heteroatom-doped functional carbon nanomaterials.^11^ For instance, the pyrolysis of bread or wheat flour comprising metal precursors has been used to produce activated carbon foams^8^ or other carbon materials with functional nanoparticles (Fe, Mn, Co and TiO_2_).^3,12,13^ In the specific case of bread, its spongelike structure seems particularly suitable for incorporating the metal precursors by impregnation in solutions, and easily obtaining after dying and pyrolysis a composite of porous carbon with a homogeneous distribution of metal nanoparticles.

The catalytic properties of silver have long been known. More than a century ago, it was found that crystalline Ag catalyzed the oxidation and dehydrogenation of methanol into formaldehyde and this process is still exploited by BASF company in the industrial production of this chemical.^14^ Recent research is still focused on the potential application of Ag nanoparticles (NPs) as catalysts for a broad range of chemical reactions.^15,16^ Similarly it has been long known that Ag NPs present electrocatalytic activity toward the cleavage of halide bonds.^17^ In this context, Ag NPs combined with carbon materials have been studied combined with electrochemical techniques for the removal^18^ and analysis^19,20^ of hazardous chlorinated compounds. Similarly, the combination of electrochemical techniques and sensors based on carbon materials with Ag NPs is highly appealing for the detection of halogenated compounds. Carbon materials have been widely used in electrochemistry owing to their chemical inertness, robustness and wide potential windows. The integration of Ag NPs in carbon structures can be applied to the reduction of organic halides at small negative potentials, well above those inducing the hydrogen evolution reaction. Then, the halide anions released into the solution can be measured since at low positive potentials the oxidation of Ag NPs to form Ag halides gives rise to an anodic current.^21−23^

Halide ions are present in many industrial and environmental processes, being in some cases of widespread use such as in the deicing of roads and disinfection of tap water.^23−25^ To avoid environmental and health impacts, it is necessary to avoid excessive or deficient concentration levels of halides in natural waters.^26^ In fact, the U.S. Environmental Protection Agency (EPA) standard for chloride concentration in drinking water is 250 ppm (1 mM = 35.5 ppm).^27,28^ In addition, the EPA indicated that permitting authorities may set water quality-based effluent limitations on bromide to protect downstream drinking water plants and their customers from the effects of increasing concentrations in source waters.^27,29^ More specifically, halogenated organic compounds are relevant hazardous chemicals for their persistence, toxicity and complex chemistry in the environment.^30,31^ In this context, electrochemical sensors could provide simple and cost-effective methods for analyzing those chemicals in the water.

The purpose of this study is to evaluate the potential of Ag/C nanocomposite materials prepared from bread waste as functional materials for the electrochemical analysis of halide anions (Cl^–^, Br^–^, I^–^) and halogenated organic compounds (sucralose and trichloroacetic acid) in waters. Sucralose is an artificial sweetener commonly used as a sugar substitute by the food industry and is poorly degraded during conventional wastewater treatments. It is considered a contaminant of emerging concern.^32,33^ Some reports indicate that the detection of sucralose can be used as an indicator of anthropogenic discharge and water pollution events.^34,35^ Trichloroacetic acid (TCA) has carcinogenic and mutagenic properties and is a nonbiodegradable disinfection byproduct found in chlorinated drinking water systems, agriculture and cosmetic industry effluents. Thus, TCA is considered a major environmental issue and should be monitored in waters.^36,37^

In this work, we developed a Ag/C nanocomposite material produced by impregnating a dried bread waste with a silver precursor solution and further carrying out pyrolysis at high temperature. Carbon paste electrodes (CPE) were prepared with the Ag/C nanocomposite and the analytical performance for analyzing halides (Cl^–^, Br^–^ and I^–^), sucralose and trichloroacetic acid were thoroughly assessed.

## Experiment

### Reagents and Solutions

All reagents were of high purity, analytical grade, or equivalent. Silver nitrate (AgNO_3_, AppliChem GmbH) and spectroscopic grade liquid paraffin (Uvasol from Merck) were used as received. Ortho-phosphoric acid (0.1 M) (85%, Sigma-Aldrich) was used to prepare phosphate buffer (PB) solution. Potassium chloride (KCl, 99+%,), potassium bromide (KBr, 99+%), potassium iodide (KI, 99.5+%), sodium hydroxide (NaOH), trichloroacetic acid (99+%, Cl_3_CCOOH) and Sucralose (98+%) from Sigma-Aldrich were used as received. Salt-free bread was bought from a local supermarket, with a salt content of 0.05 g/100 g (see the bread compositional information in Table S1 (Supporting Information, SI)). The bread was placed in a cool and dry place for 2 weeks and then used for the experiments.

### Preparation of Nanocomposites

The preparation of Ag/C nanocomposites comprised the impregnation of the bread in deionized water containing silver nitrate, at a mass ratio of silver nitrate: water: bread of 1:59:15. After impregnation for 5 min, the mixture was fully stirred to obtain a slurry-like structure. Thereafter, a 1 cm thick layer of the mixture was poured onto an evaporating dish, which was placed in a stove at 60 °C for 2 days to induce evaporation of water. The resulting dry material was pyrolyzed at 1050 °C in an Ar atmosphere for 2 h. The Ag/C nanocomposite material that was formed was then ball milled in a Retsch Mixer Mill MM 400 for 10 min at 15 Hz, using a 10-ml zirconia jar and two 12 mm diameter zirconia balls to obtain a powder with an average particle size of 12 μm (Figure S1, Supporting Information). A pure C material was also prepared for comparison purposes following the same procedure described above but without carrying out the impregnation in the silver nitrate solution.

### Preparation of Carbon Paste Electrodes of Ag/C Materials (CPE_Ag/C_)

Carbon paste electrodes are convenient to characterize the electrochemical performance of different materials.^38,39^ Here, 500 mg of Ag/C nanocomposite powder and 0.16 mL of liquid paraffin were mixed thoroughly in a mortar to prepare a carbon paste. This paste was packed into a 3 mm diameter well, defined at one end of a 6 mm diameter Teflon body, into which a 3 mm diameter stainless steel rod was inserted to make the electrical contact. The surface of the prepared carbon paste electrode was manually polished on an A4 paper. Following one analysis, the carbon paste was discarded and a new carbon paste electrode was made for the following analysis.

### Characterization of the Ag/C Materials

Morphological characterization of the materials was carried out using a scanning electron microscope (SEM), Auriga from Carl Zeiss, operated at 5–10 kV also equipped with an Energy Dispersive Spectroscopy analysis (EDS) detector. The SEM images were used to estimate the size distributions of Ag NPs in the composite, measuring more than 150 different particles with the ImageJ software.^40^ The particle size distribution of the Ag/C powder was analyzed by light scattering with a Mastersizer 2000 (Malvern Instruments). The nanocomposite porosity was studied by nitrogen adsorption–desorption isotherms with a Malvern Micromeritics equipment after degassing around 0.3 g of sample in a vacuum (pressure <1 mPa) for 48 h at 150 °C. Nitrogen adsorption analysis was carried out to estimate the specific surface areas by the Brunauer-Emmet-Teller (BET) method. The pore volume, pore size distributions and mean pore diameters were determined by the Barret-Joyner-Halenda (BJH) method.^41^ Powder X-ray diffraction (XRD) patterns of the nanocomposites were recorded with a Siemens D-5000 diffractometer with Bragg–Brentano geometry using Cu anode with wavelengths Cu_Kα1_ = 1.5406 Å and Cu_Kα2_ = 1.5444 Å in the 2θ range of 10–80°. The XRD data were acquired in steps of 0.05° and acquisition time of 8 s per step.

### Electrochemical Analysis

All measurements were performed at room temperature using a conventional 20-ml three-electrode electrochemical cell configuration. This included a platinum counter electrode and an Ag/AgCl reference electrode (both from Metrohm AG, Switzerland), together with the 3 mm diameter CPE_Ag/C_, described above. An Autolab PGSTAT30 potentiostat (EcoChemie, The Netherlands) was used in all the electrochemical measurements. Cyclic voltammetry (CV) and square wave voltammetry (SWV) techniques were used in all the studies described below. All measurements were performed in phosphate buffer (PB) solution. The CV curves were recorded at the scan rate of 0.1 V s^–1^. In order to obtain a stable signal and use it for a reliable quantitation, the second CV scan was used for the data processing. The SWV was recorded by applying a potential scan from −0.5 to 0.6 V (vs Ag/AgCl) with a step increment of 5 mV, an amplitude of 25 mV, frequency of 20 Hz.

## Results and Discussion

### Microstructural Characterization

**Figure 1:**
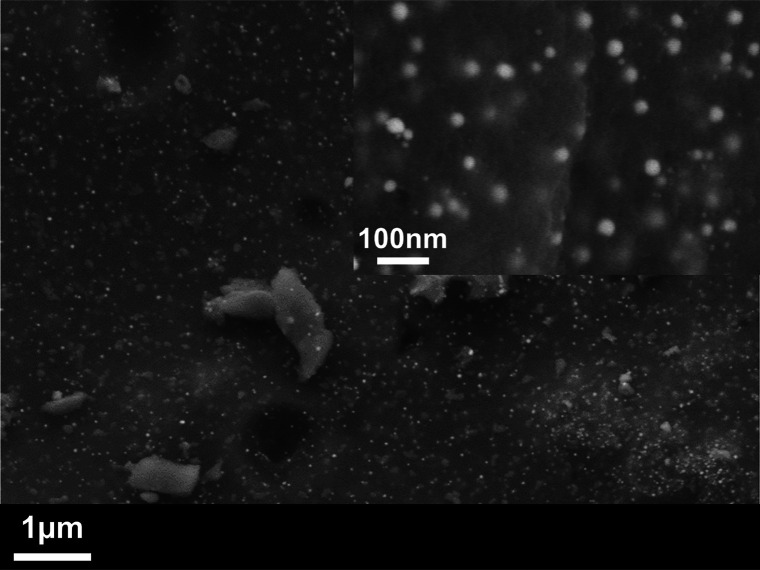
Secondary electron SEM images of Ag/C nanocomposite. The inset shows a higher magnification image of the same sample.

SEM images of the Ag/C nanocomposite recorded at different magnifications, shown in Figure 1 and Figure S2A (SI), reveal the presence of uniformly distributed small bright spherical particles on the surface of the dark matrix or embedded in it. The bright contrast of the spherical particles indicates the presence of Ag metal while the darker matrix can be attributed to carbon. Particles size distributions obtained from the SEM images indicate that the sizes of these small bright particles were in the range of 25–40 nm (Figure S2B, Supporting Information). The EDX analysis revealed a mass ratio of Ag to C of around 1:3 (Figure S3, Supporting Information).

Nitrogen adsorption and desorption isotherms of the Ag/C composite material (Figure S4A, Supporting Information) indicated the presence of micropores (<2 nm), mesopores (2–50 nm) and macropores (50–7500 nm), according to the BET model, the slope and the shape of the adsorption curve.^39,42^ The surface area of Ag/C nanocomposite was calculated to be 11.58 ± 0.03 m^2^·g^–1^ (Table S2, Supporting Information) with a total pore volume of 0.015 ± 0.002 cm^3^·g^–1^ according to the nitrogen adsorption at a relative pressure *P*/*P*_0_ of about 0.995. The BJH pore size distribution curve obtained from the adsorption isotherm confirmed the existence of pores mostly in the micropore and mesopore scale with a small proportion of macropores. From the characterization of the porosity of pure C made from bread waste, a surface area of 8.55 ± 0.03 m^2^g^–1^ and a total pore volume of 0.012 ± 0.001 cm^3^g^–1^ were obtained (Figure S4B, Table S2, Supporting Information). The incorporation of silver particles slightly increases the porosity of the composite, which has a positive effect on the targeted application of the material as the electrochemical working electrode.

**Figure 2:**
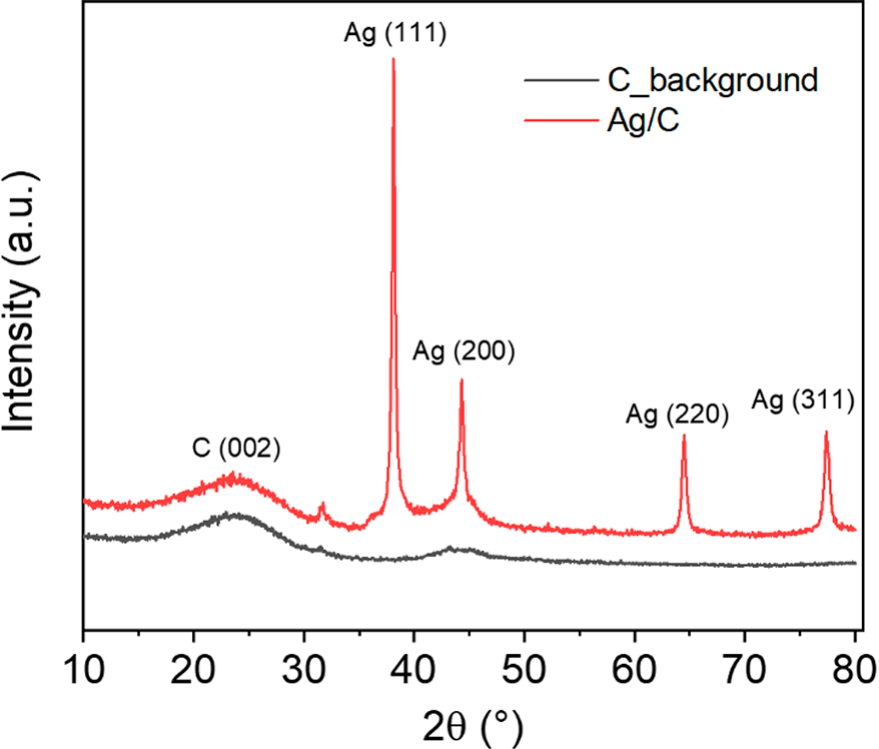
X-ray diffraction patterns of the pure C and Ag/C composite.

The Ag/C nanocomposite and the pure C material were also studied by XRD (Figure 2). For both materials, the diffractograms present a broad bump at around 2θ = 23.6°, which corresponds to the scattering induced by the amorphous carbon. In the pattern of Ag/C composite, the four peaks located at 38.1°, 44.4°, 64.6°, and 77.4° can be respectively assigned to the (111), (200), (220), and (311) planes of the face-centered cubic Ag crystalline structure. This confirms that the nanoparticles obtained during the pyrolysis consist of crystalline metallic silver.

### Electrochemical Characterization and Analysis of Halides in Water

**Figure 3:**
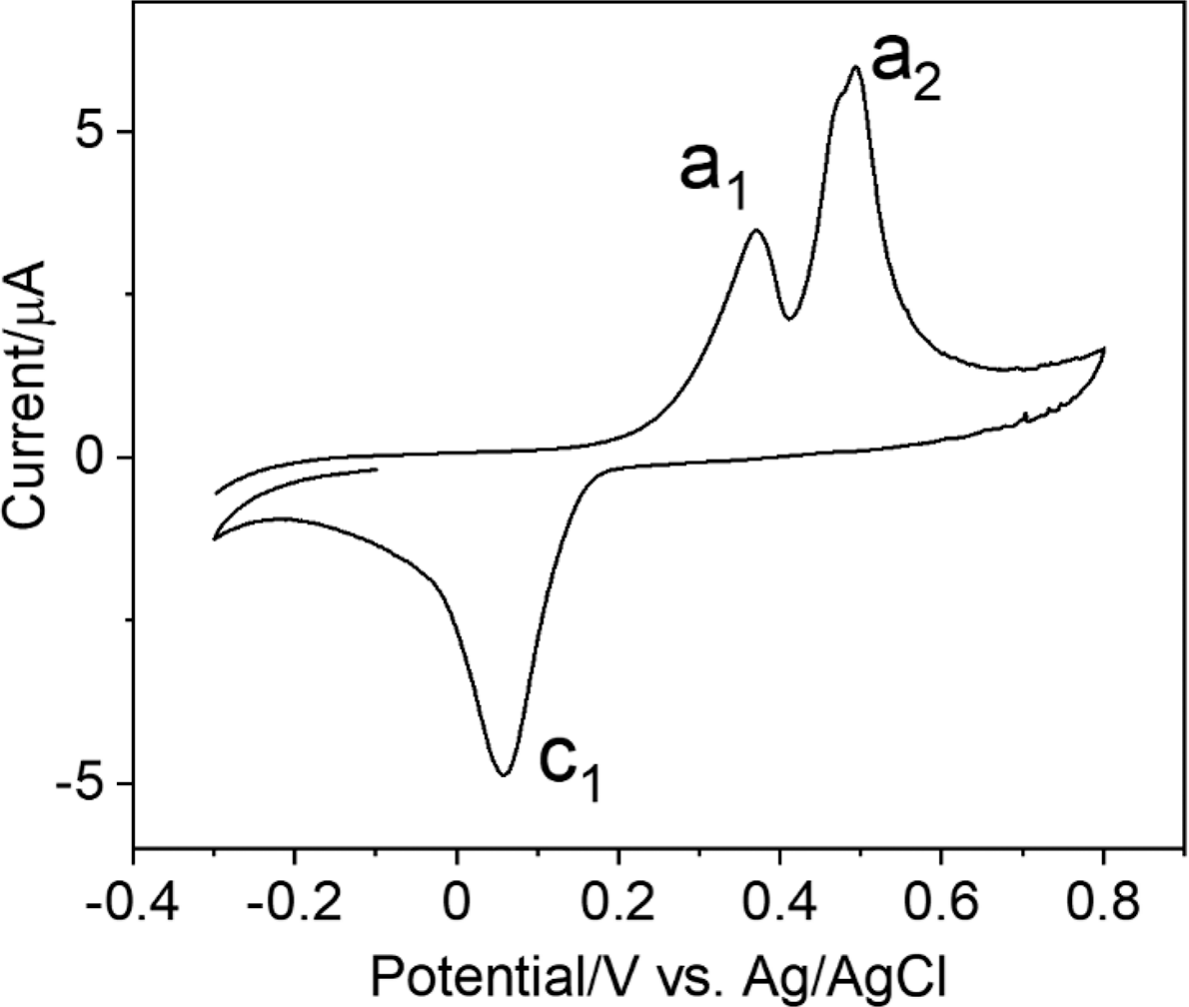
Cyclic voltammogram in PB solution (pH = 6.0) using the CPE_Ag/C_ at scan rate of 100 mVs^–1^.

As shown in Figure 3, a cyclic voltammogram (CV) with the CPE_Ag/C_ was recorded in PB background solution (pH = 6.0) to visualize the electrodic redox process that the Ag NPs underwent. Two anodic peaks at 0.37 V (a_1_) and 0.49 V (a_2_), and one cathodic peak centered at around 0 V (c_1_) were recorded. The a_1_ and a_2_ can be ascribed to the formation of Ag_2_O and AgO, respectively, in agreement with the literature.^43−45^ The c_1_ may be related to the reduction of the Ag oxides and the formation of Ag^0^ species on the electrode surface.^43−45^Figure S5 shows the different CV scans using the produced CPE_Ag/C_. After 10 scans, the peak current value keeps 95% of that of the 2nd scanning cycle indicating our CPE_Ag/C_ electrode is electrochemically stable.

**Figure 4:**
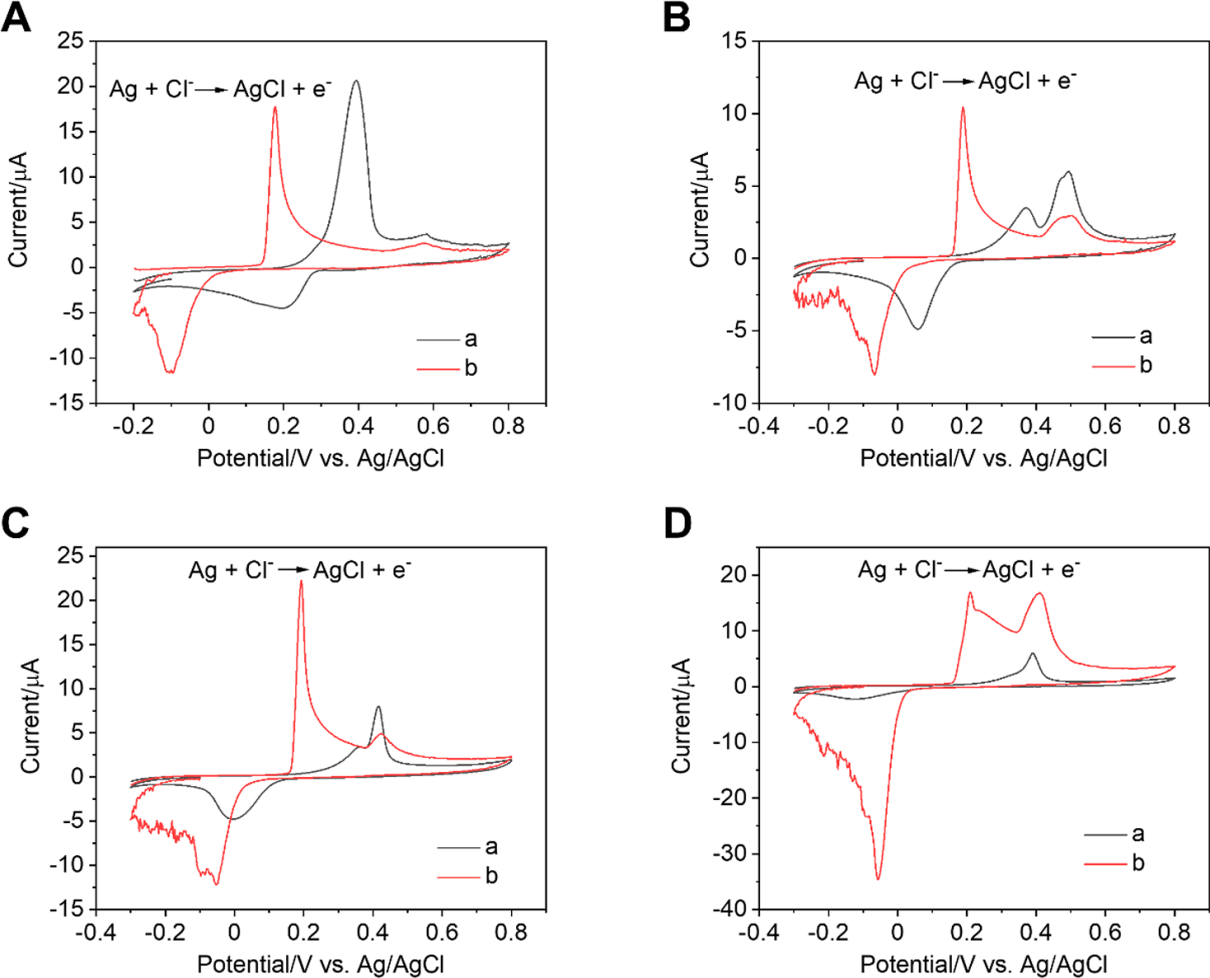
Cyclic voltammograms recorded with the CPE_Ag/C_ in PB solutions in the absence of Cl^–^ (a) and presence of 5 mM Cl^–^ (b) at different pHs. (A) pH = 1.6, (B) pH = 6.0, (C) pH = 7.5, (D) pH = 9.6. Scan rate = 100 mV s^–1^.

The performance of CPE_Ag/C_ was initially studied in the detection of halide ions. Figure 4 shows the CVs recorded in PB solutions of different pHs containing 0 and 5 mM of Cl^–^. The oxidation peaks at around 0.4 and 0.5 V which appear in the CVs obtained without Cl^–^ (black curves) for most of the studied pH conditions can be ascribed to the oxidation of Ag (0) to Ag(I) and Ag(II), respectively.^43^ In the presence of Cl^–^ (red curves), the oxidation peak at around 0.2 V is due to the reaction between Ag and Cl^–^.^22,46^ The peaks related to the formation of Ag oxides became smaller when Cl^–^ was added to the electrolyte at pH 1.6, 6.0, and 7.5. This is because the formation of AgCl took place at lower anodic overpotentials in the anodic potential scan and is thus the predominant electronic process.^23^ At pH of 9.6, both the formation of AgCl and Ag oxides give rise to overlapped signals, with the latter being of high current intensity. This is likely due to fact that the alkaline condition favors the formation of Ag oxides.^43,45^ From these measurements the detection of Cl^–^ appears to be more suitable at pH values of 6.0 and 7.5. At pH 1.6, the electrolyte is strongly acidic, while at pH of 9.6, the oxidation peaks of Ag (0) to Ag oxides are so significant that may interfere with the peak related to the formation of silver chloride. Similar studies were carried out for the detection of Br^–^ and I^–^ ions and the recorded voltammograms are shown in Figures S6 and S7 (SI). Anodic peaks at 0.07 and −0.15 V, which could be ascribed to the formation of AgBr and AgI, respectively, were recorded. The relative peak current values related to the formation of the corresponding silver halides and silver oxides were also observed similarly to what was found for Cl^–^. In order to avoid the interference of the AgO formation, a PB solution at pH 6 was selected as the background electrolyte for all the following studies.

Pure carbon materials have previously shown good electrochemical/electrocatalytic properties when working under specific experimental conditions.^47,48^ Thus, we studied the response of CPEs made of pure carbon from pyrolyzed bread in solutions containing the target halides (Figure S8, Supporting Information). When the concentration of Cl^–^ and Br^–^ was above 4.5 mM, very small oxidation peaks appeared at around +0.20 V and +0.35 V, respectively. For I^–^, no oxidation peak was detected in the CVs in the tested concentration range. These results indicate that the carbon matrix does not contribute to the halide detection and just performs as the electrode conductive base material.

**Figure 5:**
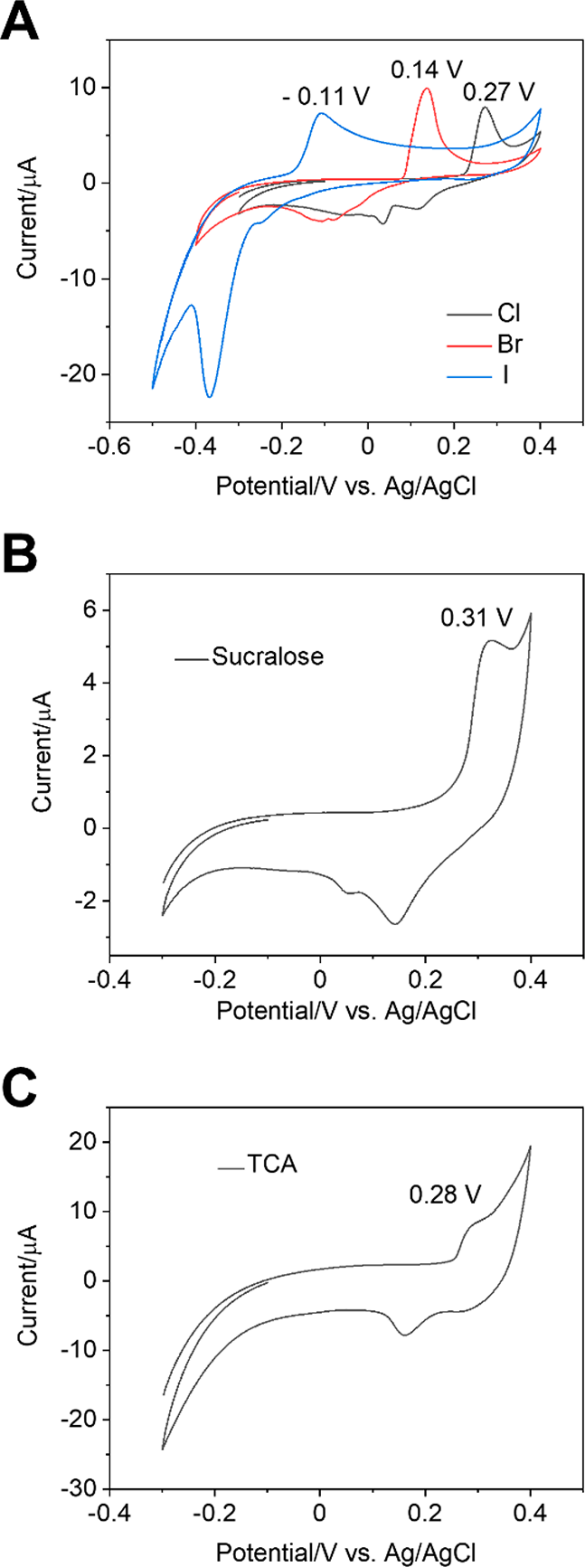
(A) Cyclic voltammograms obtained with the CPE_Ag/C_ in PB solution (pH = 6) containing 395 μM chloride, bromide, and iodide, respectively, in a separated solution, (B) cyclic voltammogram recorded with the CPE_Ag/C_ in PB solution containing 9 mM sucralose, (C) cyclic voltammogram recorded with the CPE_Ag/C_ in PB solution containing 5 mM trichloroacetic acid. The scan rate is 100 mV/s.

Figure 5A shows the cyclic voltammetric detection of the three halide ions using the CPE_Ag/C_. Well-defined anodic peaks at potentials of 0.27, 0.14, and −0.11 V (vs Ag/AgCl) were recorded, which can be respectively ascribed to Cl^–^, Br^–^ and I^–^. A silver halide precipitate forms on the surface of the Ag NPs and consequently generates an oxidation current on the voltammogram, which is in line with the equation of Ag + X^–^ → AgX + e^–^ (X = Cl^–^, Br^–^ and I^–^).^21,22^ The well-distinguished peak potentials, which do not overlap for the different halides can be related to the different solubility products (*K*_sp_) of these species, which determines thermodynamically the interaction tendency between the surface silver atoms and the halide anions.^22,46^ The degree of difficulty to form silver compounds (e.g., AgCl, AgBr and AgI) through the formation of Ag-anion bonds would affect the oxidation/reduction potential. It is worth noting that the *K*_sp_ order of silver compounds in water is Cl^–^ > Br^–^ > I^–^ (*K_sp_* for AgCl = 1.76 × 10^–10^, for AgBr = 5.32 × 10^–13^ and for AgI = 8.49 × 10^–17^).^22^ The smaller the *K_sp_*, the stronger the chemical affinity between the silver surface atoms and the halide anions, thus the significant *K_sp_* differences between the different halides contribute to their well-separated oxidation potentials.

Figure S9A presents the voltammetric responses of Cl^–^ solutions of increasing concentrations in PB and Figure S9B is a zoomed-in image of the oxidation peaks recorded between 0.2 and 0.4 V. As expected, the peak intensity increases with the Cl^–^ concentration. The calibration curve obtained by plotting the peak current vs the Cl^–^ concentration (Figure S9C) shows a linear response albeit with a decreased slope for concentrations above 500 μM. The limit of detection is 16 μM estimated using 3σ IUPAC criterion. This value is below the maximum allowable concentration of 250 ppm (∼7 mM) in drinking water set by the USA EPA.^27,28^ Similar studies were carried out for Br^–^ and I^–^ (Figures S10 and S11, Supporting Information). The oxidation peaks shift to lower potentials with increasing concentrations of halide ions as expected from the Nernst equation.^49,50^ Two linear ranges were also estimated in the detection of both species, showing higher sensitivity at lower analyte concentrations. The decreased sensitivity above a critical concentration could be indicative of Langmuir type adsorption of halides at the electrode surface.^51,52^ Similar behaviors were reported for the electrochemical analysis of other chemical species.^51,53^ The analytical parameters obtained from the calibration curves of the different halides are summarized in Table 1.

**Table 1:** Analytical Parameters Obtained from the Calibration Curves of CPE_Ag/C_ for Halides, Sucralose, and TCA Analysis^a^

Sensor	Analyte	Method	Slope·10^3^ (μA μM ^–1^)	Intercept (μA)	*R*^2^ (*n* = 3)	LOD (μM)	Linear range (μM)
CPE_Ag/C_	Cl^–^	CV	9.1 ± 0.3	4.1 ± 0.1	0.993	16	50–491
			6.5 ± 0.2	5.3 ± 0.2	0.995		491–1983
	Br^–^	CV	21.8 ± 0.9	0.6 ± 0.08	0.993	8	20–491
			2.7 ± 0.1	8.7 ± 0.2	0.988		491–4295
	I^–^	CV	21 ± 1.4	0.5 ± 0.06	0.992	7	10–149
			12.5 ± 0.1	1.7 ± 0.07	0.998		149–2318
	Sucralose	CV	1.2 ± 0.2	2.6 ± 0.1	0.946	141	200–990
			0.18 ± 0.005	3.5 ± 0.04	0.995		990–16700
	TCA	CV	1.15 ± 0.03	2.5 ± 0.1	0.996	326	3114–5508
		SWV	1.62 ± 0.06	0.9 ± 0.09	0.994	167	395–3114

a LOD is calculated using the 3σ IUPAC criterion.

Figure S12 shows cyclic voltammograms of a PB solution containing the same concentration of Cl^–^, Br^–^ and I^–^. The peak potentials are well separated and in agreement with those observed in Figure 5, as well as in Figures S9, S10, and S11 (SI). This allows those ions to be simultaneously identified because the *K_sp_* for the formation of the different silver halides in water differs by a few orders of magnitude. In this regard, it is not surprising that the largest peak current is associated with the oxidation of I^–^, the anion more prone to form a silver halide. It is also worth mentioning that while the oxidation peaks for Br^–^ and I^–^ are already visible from the second cycle, the Cl^–^ peak only appears after several cycles. This can result from the competition between the different halide anions for interacting with the surface of Ag NPs. Initially, there is the same concentration of Cl^–^, Br^–^ and I^–^ around the silver NP surfaces but due to the stronger interaction of I^–^ and Br^–^ with the silver surface atoms, the first to appear are the oxidation peaks of Br^–^ and I^–^. However, after several scans, the concentration of Cl^–^, becomes comparatively larger than that of the already depleted Br^–^ and I^–^, and thus the oxidation peak for Cl^–^ becomes visible.

### Analysis of Organohalide Molecules in Water

Encouraged by the positive analytical response of Ag/C nanocomposites for the analysis of halide anions reported in the previous sections, we decided to assess their potential for analyzing organohalide molecules that have been identified to be water pollutants. The electrocatalytic activity of Ag NPs for inducing the cleavage of carbon-halide bonds in this family of molecules has been previously reported^18^ and this could be exploited to electrochemically detect different target molecules following the same experimental procedure as above. Two organohalides were chosen: sucralose and trichloroacetic acid.

#### Sucralose Detection

Figure 5B shows the electrochemical response curve shape for measuring sucralose using the CPE_Ag/C_ electrode and the CVs recorded in PB solutions containing sucralose concentrations in the range from 0.2 to 16.7 mM are presented in Figure S13A,B. The peak current intensity increased with the concentrations of sucralose, showing a linear dependence in the studied concentration range. As for the calibration curves obtained for the halide ions, two distinct slopes were obtained (Figure S13C), which again may be indicative of the Langmuir type adsorption of sucralose at the surface of the electrode.^51,52^ The analytical parameters related to the calibration curves are displayed in Table 1.

**Figure 6:**
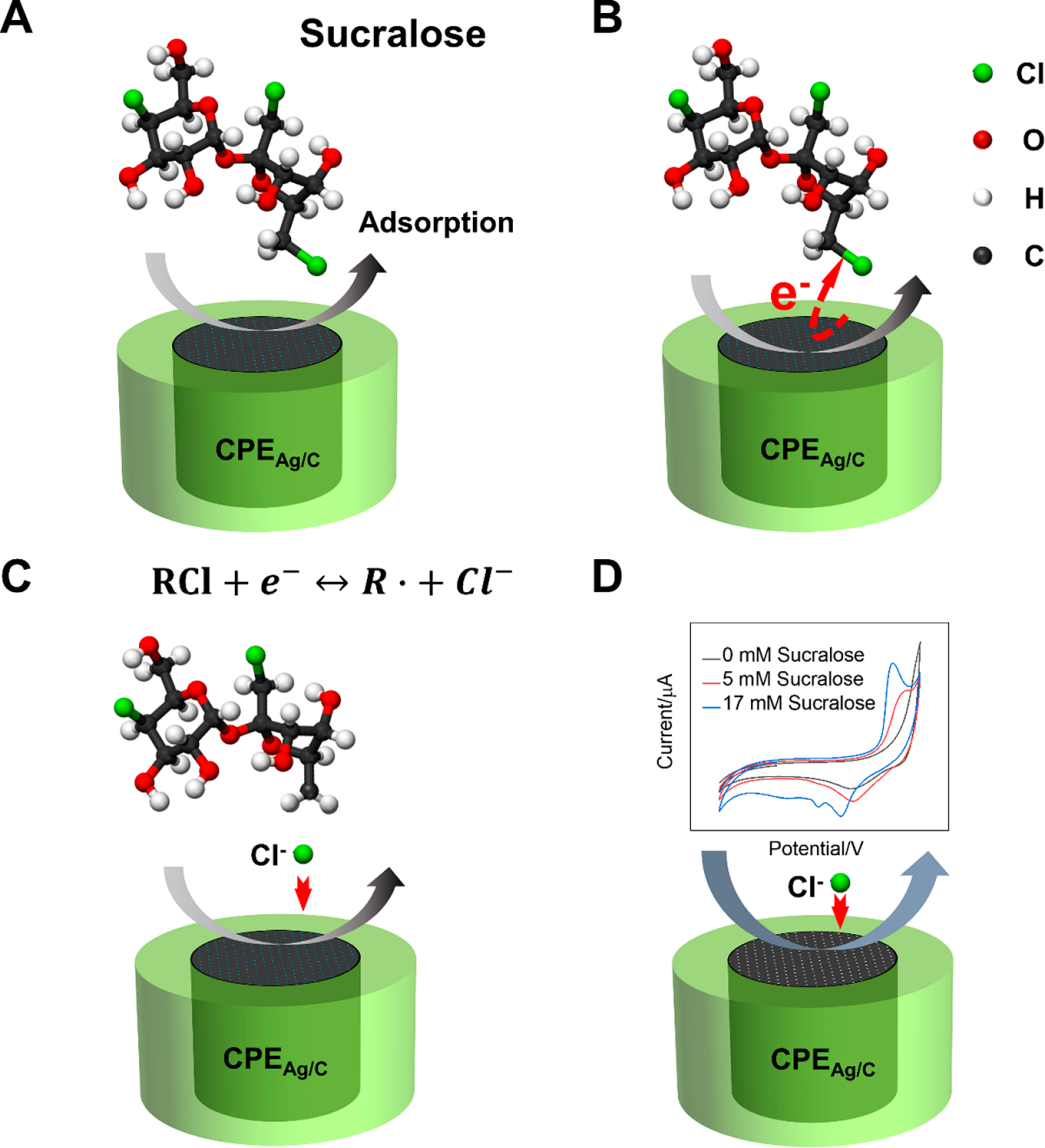
A schematic diagram of the possible underlying mechanisms in the electrochemical analysis of sucralose with CPE_Ag/C_. (A) Adsorption of the sucralose on the surface of the electrode; (B) the cleavage of the C–Cl bond and the transfer of electrons; (C) the release of free ion Cl^–^; (D) the reaction between free ion Cl^–^ and Ag NPs and the resulting analytical signal.

We conjecture that the mechanism governing the electrochemical detection of sucralose with the Ag/C nanocomposite comprises two steps, namely, the initial dehalogenation of the sucralose molecule followed by the formation of AgCl.^18,22,23^ Dehalogenation processes catalyzed by silver have been widely investigated.^54−56^ These are known to proceed by the adsorption on the surface of Ag of the halogenated compound “RX” (where R is an organic radical and X a halide), producing an “attenuated radical” intermediate of the kind R···X···Ag (see Figure 6A).^54^ The strong interaction of RCl with Ag NPs improves the kinetics of the dehalogenation reaction because the C–Cl bond is greatly weakened due to the Cl···Ag and R···Ag interactions, facilitating the cleavage of the C–Cl bond and the transfer of electrons (see Figure 6B).^18,56^ After the dehalogenation process (Figure 6C), the free ion Cl^–^ is released and it can adsorb onto the surface of Ag NPs. Then, the free ion Cl^–^ can react with Ag to form AgCl, releasing an electron which is collected by the Ag/C electrode and contributes to the analytical signal (Figure 6D) in an analogous process as for the case of halide anions in solution.

One sucralose molecule contains three Cl atoms, while the LOD for measuring sucralose is 141 μM (∼5 ppm). This high LOD, significantly larger than the one obtained for the detection of Cl^–^ in solutions (16 μM), could be due to a partial release of Cl^–^ from the sucralose molecules through the dehalogenation processes discussed above.

### Trichloroacetic Acid Determination

To further assess the performance of the CPE_Ag/C_ electrodes, we also carried out the analysis of trichloroacetic acid by CV. In this case, the mechanisms enabling the electrochemical analysis of TCA with a CPE_Ag/C_ should be similar to those discussed for sucralose. Figure 5C shows an example of the electrochemical response for measuring TCA using the CPE_Ag/C_ and Figure S14A,B show the cyclic voltammetric responses for solutions with increasing concentrations of TCA. Thereafter, the calibration curve (Figure S14C) was obtained and the analytical parameters were summarized in Table 1. We found that the linear range is limited and can only be obtained for high TCA concentrations, with also a high limit of detection. A similar poor response had been previously reported for this analyte when applying CV measurements,^57^ with a linear dynamic range between 2500 and 22500 μM. However, previous works have shown that by using Square Wave Voltammetry (SWV), a linear calibration curve could be obtained in lower concentration ranges.^20,58^ Therefore, we applied the SWV method and, as shown in Figure S15, the recorded SWV signals at different TCA concentrations and the corresponding calibration were acquired. The calculated analysis parameters were also displayed in Table 1.

For comparative purposes, the analytical performance of electrochemical sensors of halides and TCA previously reported in the literature are summarized in Tables S3 and S4 (SI), respectively. Compared with the sensors introduced in this work, these previously reported sensors show certain advantages in terms of analytical performance. However, most of these sensors rely on the electrodeposition or casting of nanoparticles on the surface of the electrodes. This is a sensor preparation process that is difficult to adapt to mass production and not suitable for translation to the industry. In the case of sucralose, as far as we know, only one research focused on the development of electrochemical sensors for its analysis. Nikolelis et al. reported an electrochemical device for the monitoring of sucralose, taking advantage of the interactions between the sucralose and bilayer lipid membranes. The adsorption of sucralose to the membranes generates an increase in the ionic current, which is ascribed to the alteration of electrostatic fields of the lipid membrane.^59^ However, the sensor manufacturation is very complex. Our work shows that Ag/C electrodes produced from bread waste can be applied to the electrochemical analysis of halides and different organohalide molecules in waters and the fabrication process for the sensor is very simple and upscalable. The sensor is nevertheless not selective for contaminants involving the same type of halide and, for instance, does not allow distinguishing between Cl^–^ anion and sucralose or TCA.

## Conclusion

A carbon nanocomposite consisting of porous carbon with Ag NPs was synthesized via high-temperature pyrolysis of a silver-containing dried bread paste, prepared by impregnating bread waste with a silver precursor solution. The generated functional Ag NPs were uniformly distributed in the carbon matrix. We evaluated the potential of the Ag/C nanocomposite for electrochemical sensor applications. To this aim, we tested the performance of the Ag/C material by producing simple carbon paste electrodes for analyzing halides (Cl^–^, Br^–^ and I^–^) and organohalides (sucralose and trichloroacetic acid). The achieved results indicate that waste bread can be upcycled to high-added-value materials by doping with specific nanoparticles and applied for electrochemical sensor purposes, thus contributing to the circular bioeconomy.

## Supporting Information Available

Composition of the bread, particle size distributions of the Ag NPs and Ag/C powder, N_2_ adsorption and desorption isotherms of the Ag/C and pure C composites, and additional experimental details for the electrochemical measurements (PDF)
