# Supplementary material for: Upcycling Bread Waste into a Ag-Doped Carbon Material Applied to the Detection of Halogenated Compounds in Waters
Source: ACS Appl Mater Interfaces. 2022 Aug 23;14(35):40182–90. doi: 10.1021/acsami.2c08332 (PMC9460431; doi:10.1021/acsami.2c08332)
Supplement: Supplementary file 1 — am2c08332_si_001.pdf [file am2c08332_si_001.pdf]

Supporting information

Upcycling bread waste into a Ag-doped carbon material applied to the detection of halogenated compounds in waters

Wenchao Duan[a,b], César Fernández-Sánchez[b,c*], Martí Gich[a*],

[a] *Institut de Ciència de Materials de Barcelona, ICMAB (CSIC), Campus UAB, 08193 Bellaterra, Spain.*

[b] *Institut de Microelectrònica de Barcelona, IMB-CNM (CSIC), Campus UAB, 08193 Bellaterra, Spain.*

[c] *CIBER de Bioingeniería, Biomateriales y Nanomedicina (CIBER-BBN), Jordi Girona 18-26, 08034 Barcelona, Spain*

* Corresponding authors: *cesar.fernandez@csic.es; mgich@icmab.es*

Table S1. Bread composition provided by a local supermarket.

net amount	210 g
nutritional information	average values per 100 g
energy value	1361 kJ/321 kcal
fats	1 g
saturated	0.30 g
monoinsaturats	0.12 g
polyunsaturated	0.58 g
carbohydrates	68 g
sugars	0.68 g
dietary fiber	2.7 g
protein	8.6 g
salt	0.05 g

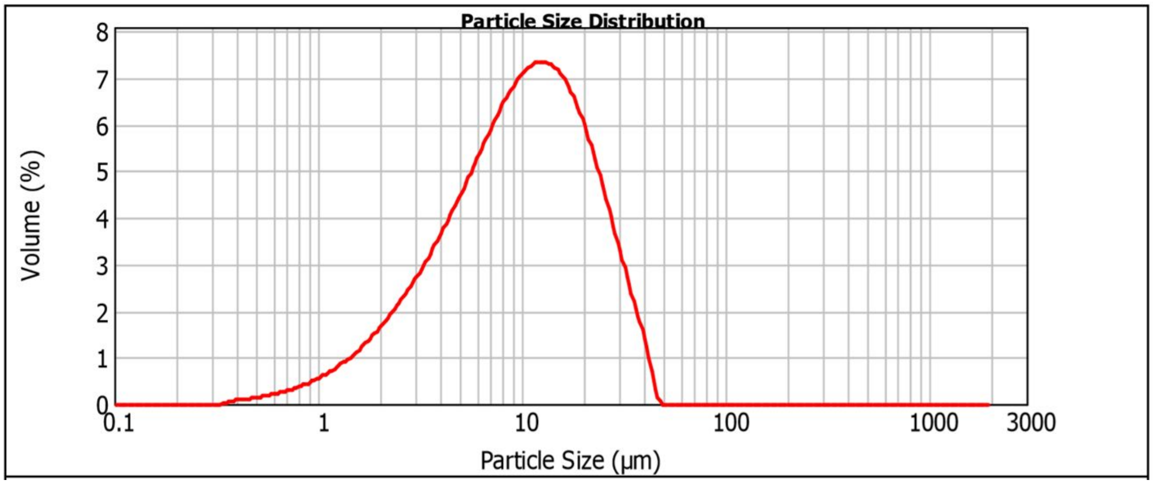

Figure S1. Graph of the particle size distribution of the Ag/C powder after ball milling.

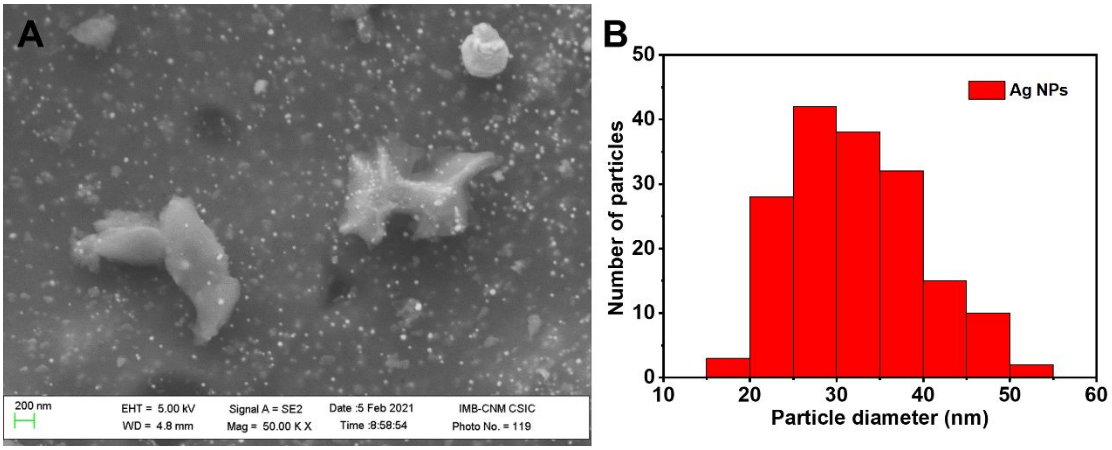

Figure S2. SEM image (A) and the particle size distribution histogram (B) of the Ag NPs in the Ag/C composite material.

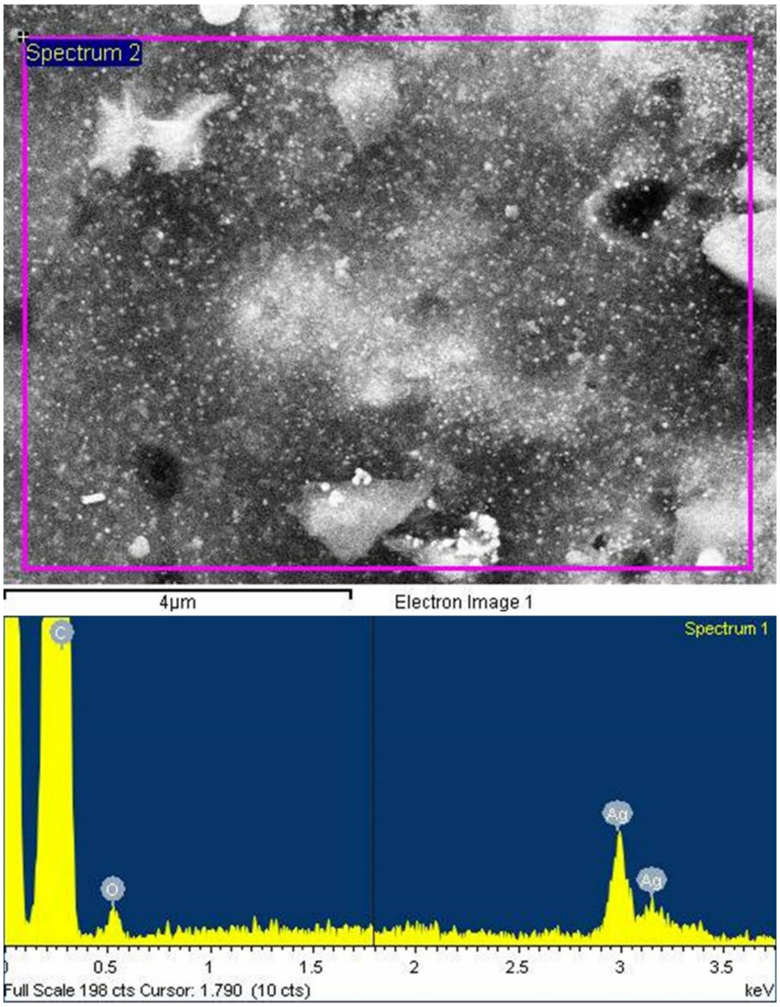

Figure S3. Energy dispersive X-ray (EDX) spectra of Ag/C composite. The EDX analysis revealed silver (Ag) and carbon (C) as the major chemical elements in the material.

element	wt. %	at. %
C	68.57	88.63
Ag	23.15	3.33
O	8.29	8.04

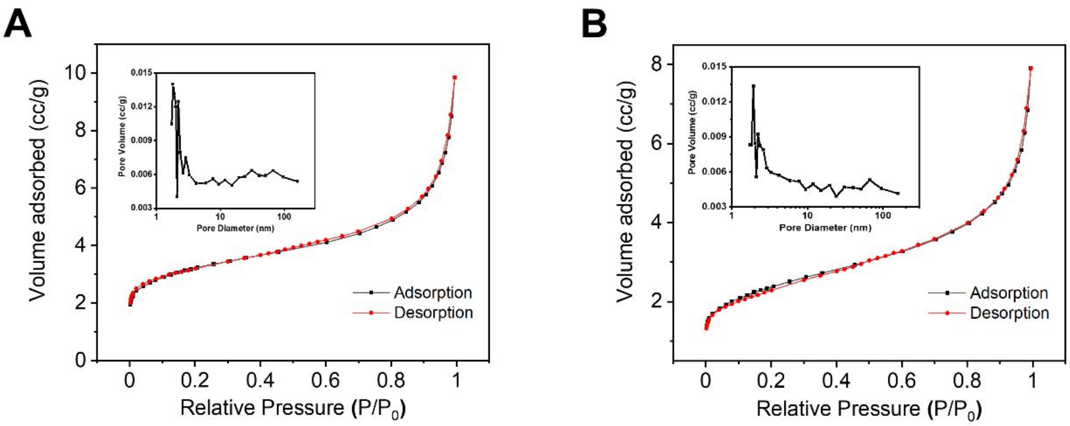

Figure S4. N_2 adsorption and desorption isotherms of the, (A) Ag/C and (B) pure C composites. Inset shows the pore size distribution determined using the BJH method.

Table S2. Textural properties of pure C and Ag/C composite made from bread.

sample	Ag/C composite	pure C
surface area (m^2g^{-1})	11.58 ± 0.03	8.55 ± 0.03
total pore volume (cm^3g^{-1})	0.015 ± 0.002	0.012 ± 0.001

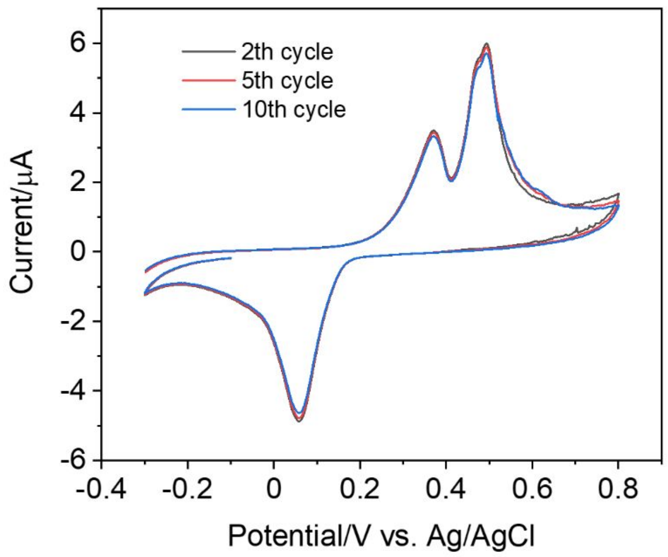

Figure S5. Cyclic voltammograms in PB solution (pH = 6.0) using the $CPE_{Ag/C}$ at scan rate of 100 mVs^{-1}.

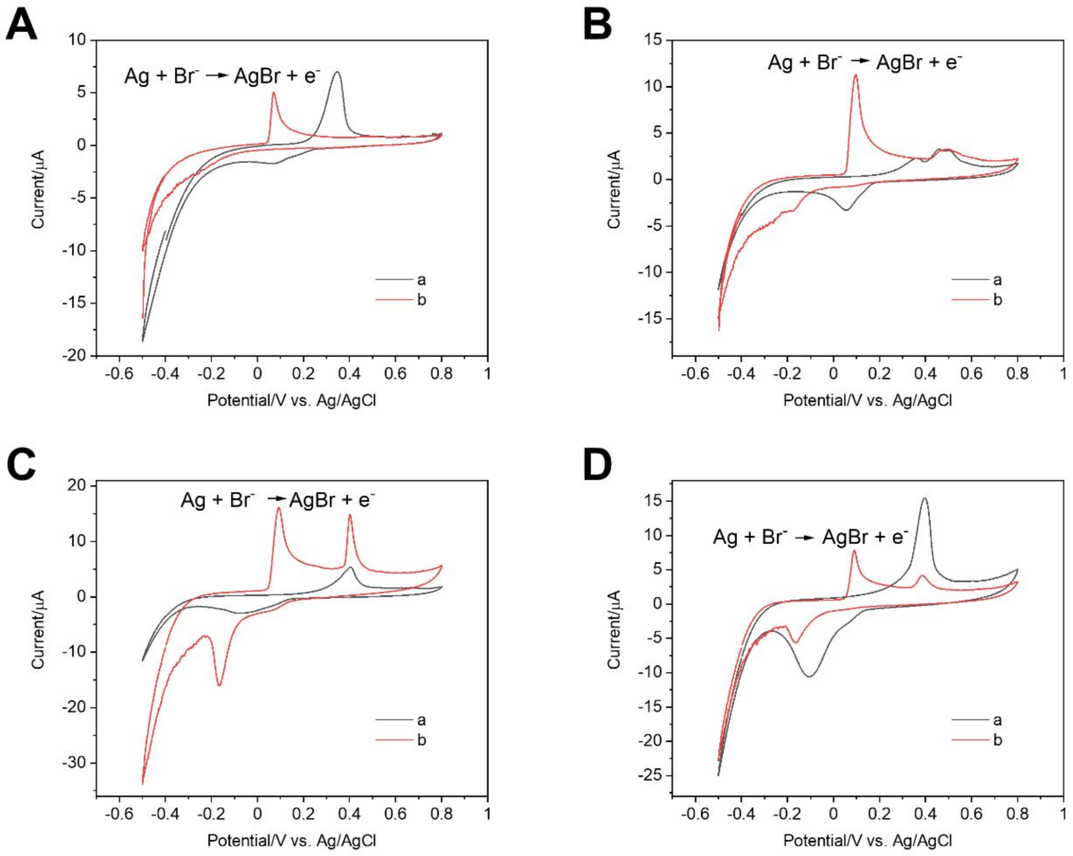

Figure S6. Cyclic voltammograms recorded with the $CPE_{Ag/C}$ in the absence of Br^- (a) and presence of 5mM Br^- (b) at different pHs. (A) pH=1.6, (B) pH=6.0, (C) pH=7.5, (D) pH=9.6. Scan rate = 100 mV s^{-1}.

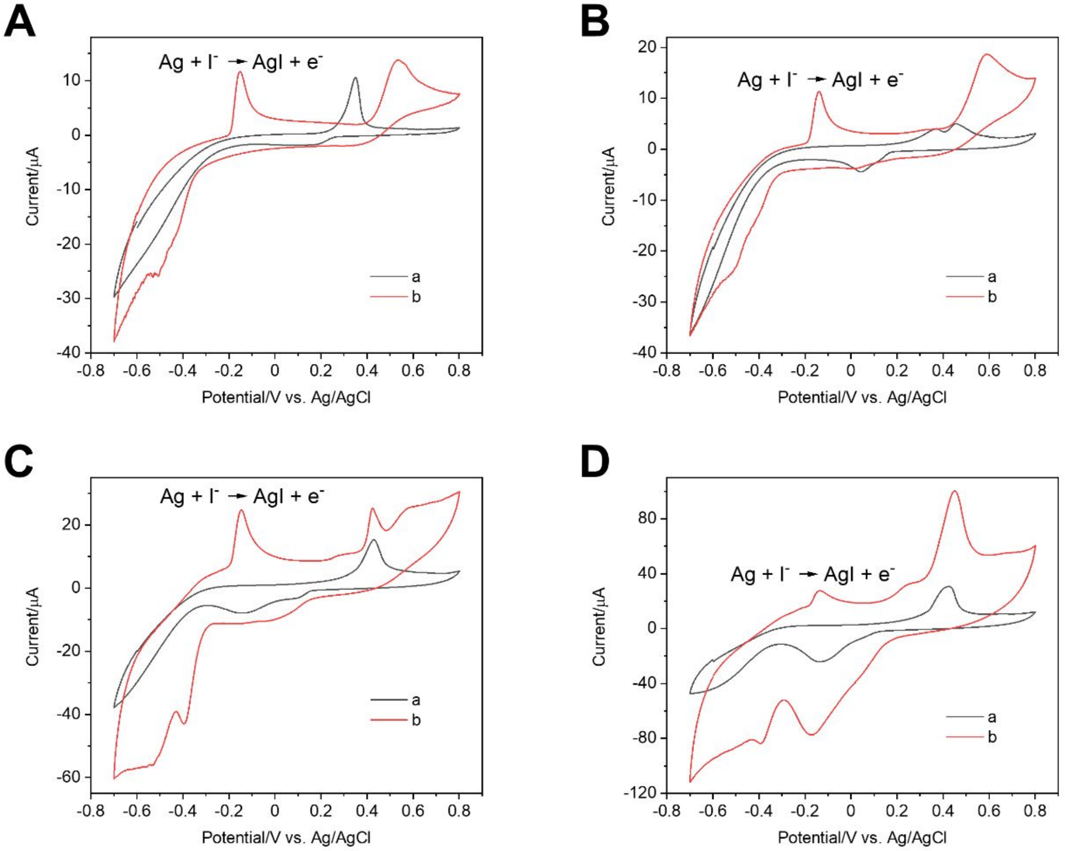

Figure S7. Cyclic voltammograms recorded with the $CPE_{Ag/C}$ in the absence of I^- (a) and presence of 5mM I^- (b) at different pHs. (A) pH=1.6, (B) pH=6.0, (C) pH=7.5, (D) pH=9.6. Scan rate = 100 mV s^{-1}.

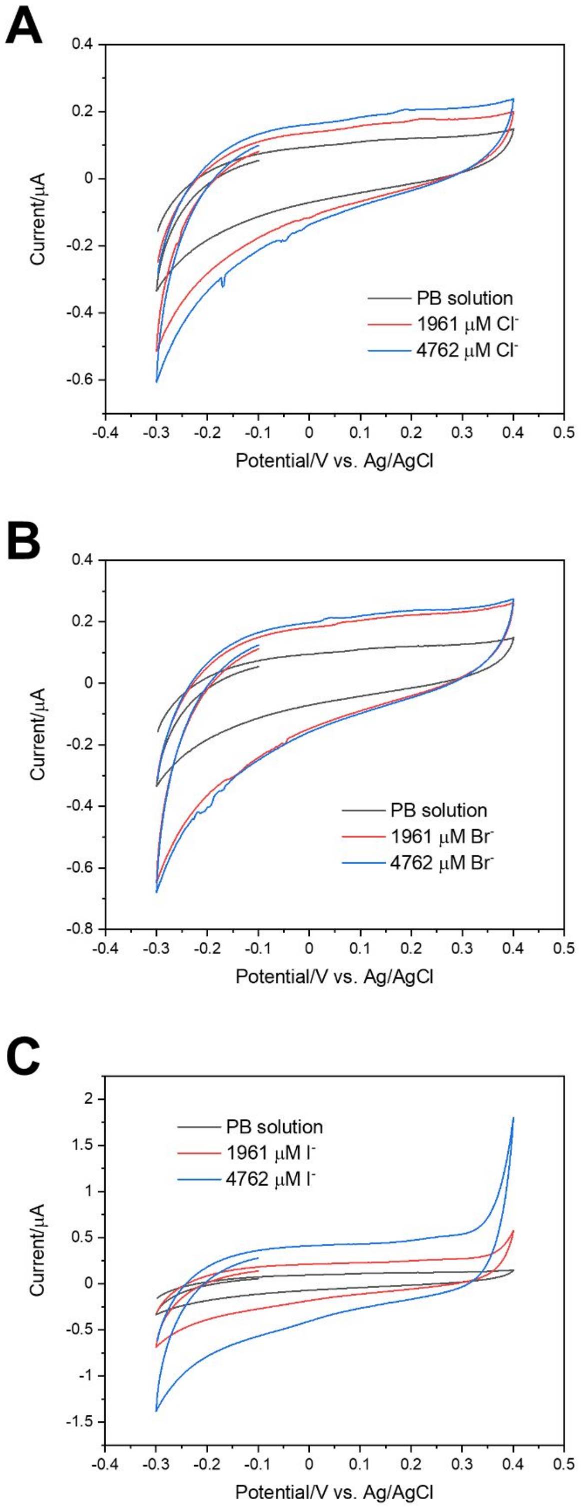

Figure S8. (A) Cyclic voltammograms recorded with the CPE made of pure C powder in PB solution (pH=6) before and after the addition of different concentrations of Cl^- (A), Br^- (B) and I^- (C). Scan rate = 100 mV s^{-1}.

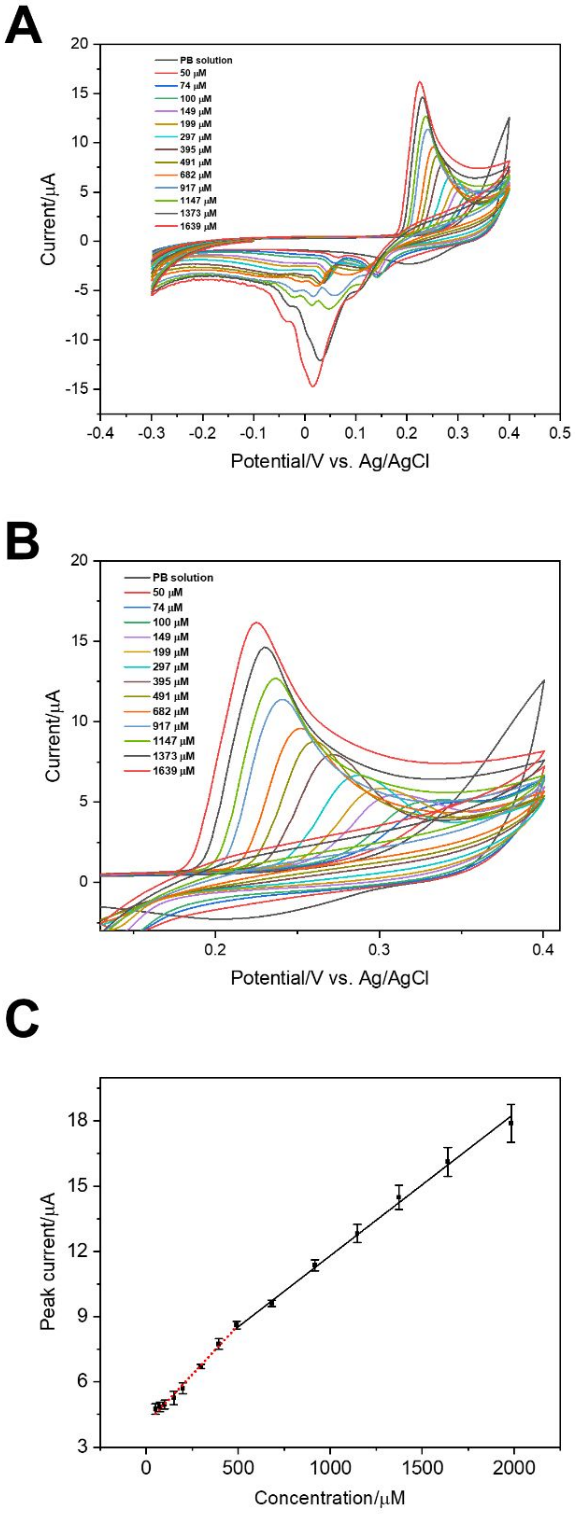

Figure S9. (A) Cyclic voltammograms of Cl^- solutions in PB of concentrations ranging from 50 μM to 1639 μM recorded with $CPE_{Ag/C}$, (B) The enlarged oxidation peak curve of cyclic voltammograms shown in (A), (C) Calibration curve of the $CPE_{Ag/C}$ for measurement of Cl^-.

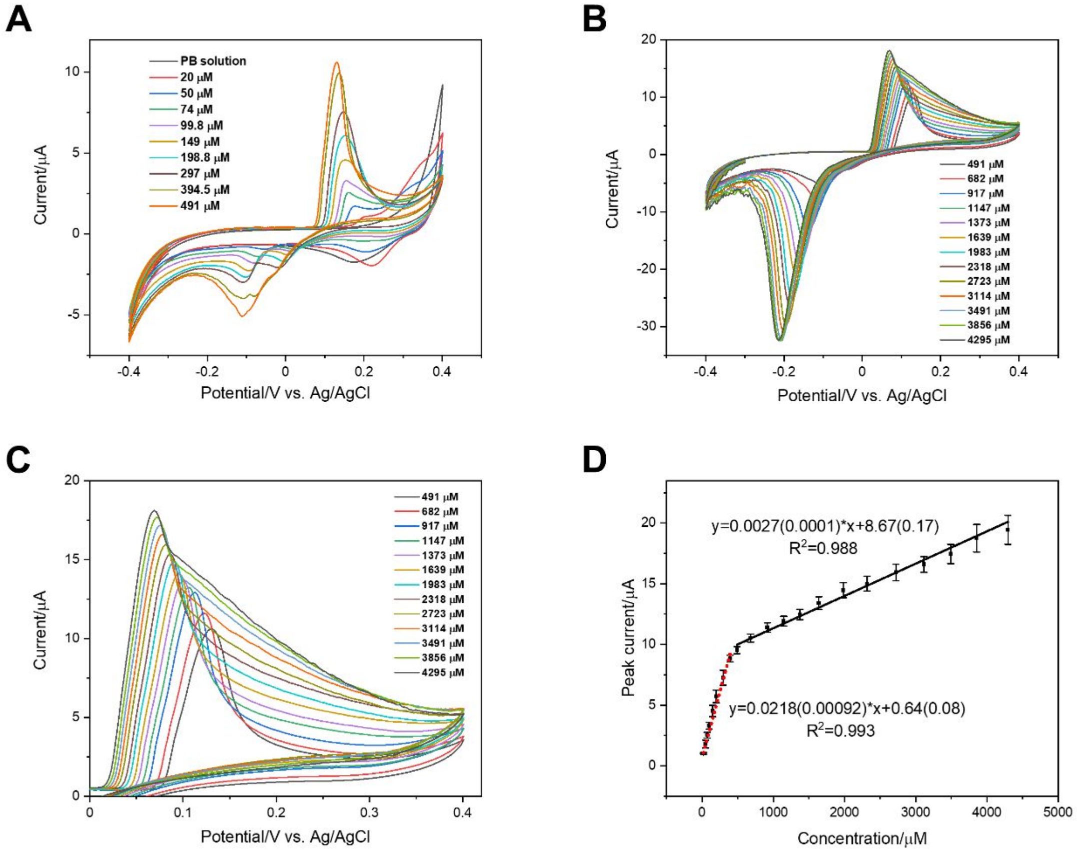

Figure S10. (A) CVs recorded with $CPE_{Ag/C}$ in PB solutions containing from 0 μM to 491 μM Br^-. (B) CVs recorded with $CPE_{Ag/C}$ in PB solutions containing from 491 μM to 4295 μM Br^-. (C) The enlarged oxidation peak curve of cyclic voltammograms shown in (B). (D) Calibration curve. Scan rate = 100 mV s^{-1}.

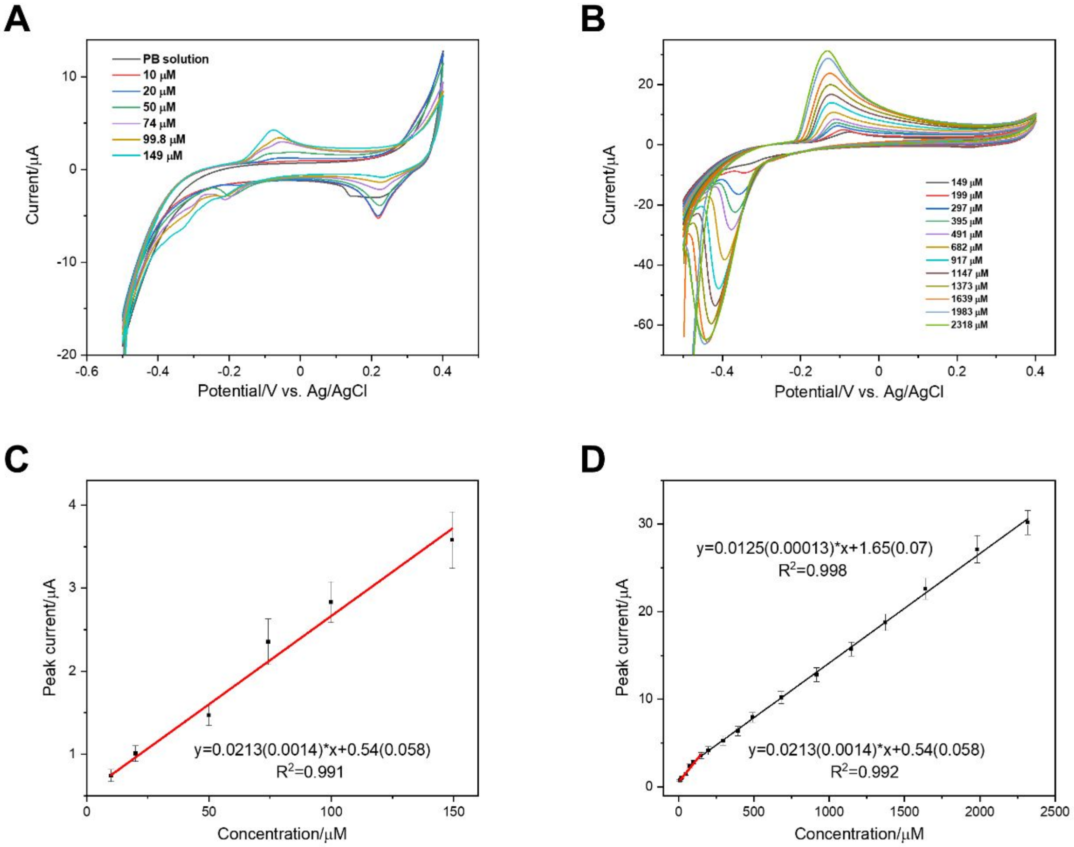

Figure S11. (A) Cyclic voltammograms recorded with $CPE_{Ag/C}$ in PB solutions containing from 0 μM to 149 μM I^-. (B) Cyclic voltammograms recorded with $CPE_{Ag/C}$ in PB solutions containing from 149 μM to 2318 μM I^-. (C) Calibration curve plotted in the I^- concentration range from 10 μM to 149 μM. (D) Calibration curve plotted in the I^- concentration range from 10 μM to 2318 μM. Scan rate = 100 mV s^{-1}.

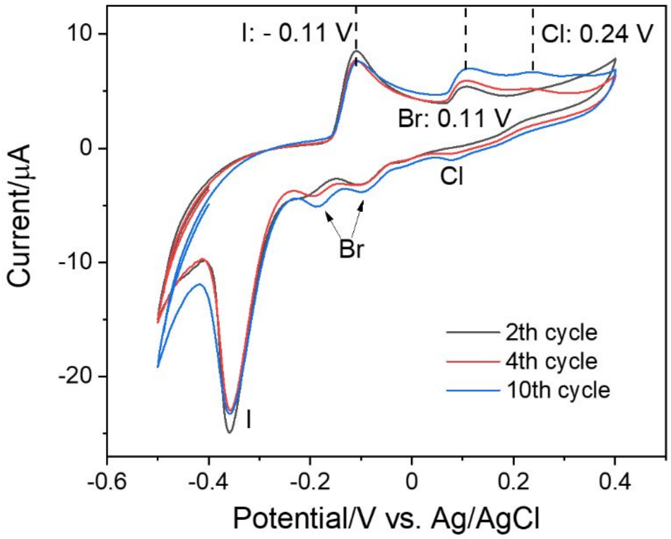

Figure S12. Cyclic voltammograms recorded with the $CPE_{Ag/C}$ in phosphate buffer solution (pH =6) containing 395 μM chloride, bromide and iodide, respectively, in a mixed solution at a scan rate of 100 mV/s.

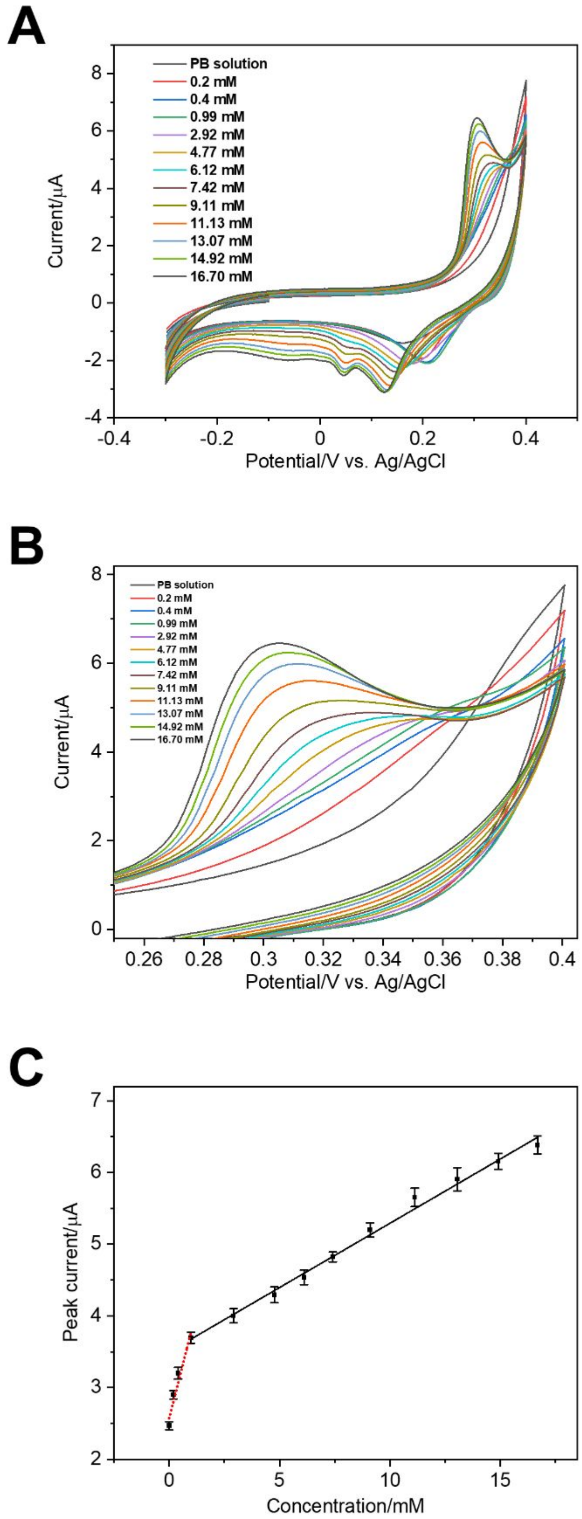

Figure S13. (A) Cyclic voltammograms recorded with $CPE_{Ag/C}$ in PB solutions containing from 0 mM to 16.7 mM sucralose. (B) The amplified peak curve of cyclic voltammograms shown in (A). (C) Calibration curve of the $CPE_{Ag/C}$ for the measurement of sucralose.

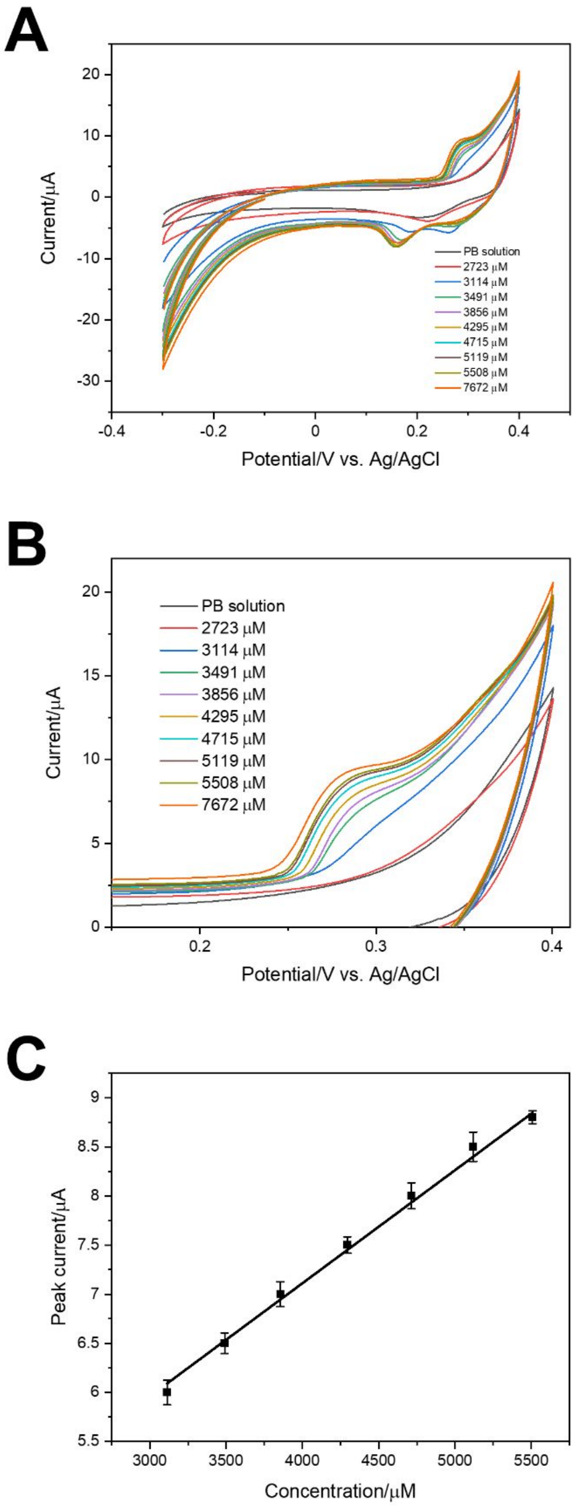

Figure S14. (A) Cyclic voltammograms recorded with $CPE_{Ag/C}$ in PB solutions containing from 0 μM to 7672 μM trichloroacetic acid. (B) Zoomed image of the CVs shown in (A). (C) Corresponding calibration curve.

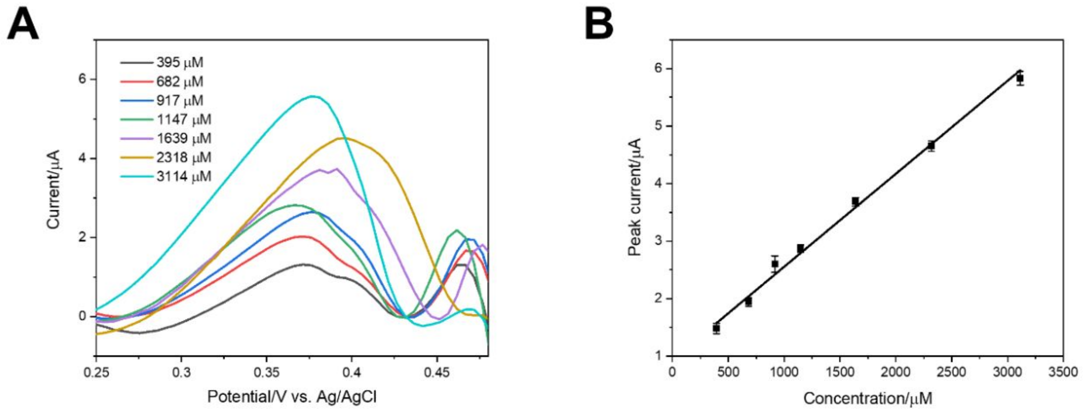

Figure S15. (A) SWVs recorded with $CPE_{Ag/C}$ in solutions containing different concentrations of TCA. (B) Corresponding calibration curve.

Table S3. Analytical parameters of the previously reported sensors for halide detection.

Modified electrodes	Halides	Linear range (μM)	LOD (μM)	References
Ag NPs-modified SPE	Cl^-	5-90	5	1
	Br^-	5-80	5	
	I^-	-	-	
Ag NWs-modified Pt electrode	Cl^-	200-20200	20	2
	Br^-	50-20200	10	
	I^-	50-20200	10	
Screen-printed silver strip sensor	Cl^-	100-20000	18.83	3
	Br^-	10-20000	2.95	
	I^-	10-20000	3.05	
Silver films on polycrystalline gold	Cl^-	0.5-20	-	4
Nano-silver modified indium-tin oxide thin electrodes	Cl^-	0.01-1	0.0052	5
Ag/dopamine modified poly(γ -glutamic acid) NPs	Cl^-	0.01-1000	0.0033	6
$CPE_{Ag/C}$	Cl^-	50-491, 491-1983	16	This work
	Br^-	20-491, 491-4295	8	
	I^-	10-149, 149-2318	7	

Table S4. Analytical parameters of previously reported sensors for TCA detection.

Modified electrodes	Linear range (μM)	LOD (μM)	References
Ag NPs-MA/GCE (SWV)	0.1-2,<br>4-100	0.030,<br>0.079	7
SNP-CS/GCE (CA)	3-56	1.1	8
Ag-MWCNT/GCE (SWV)	5-120	1.9	9
MWCNTs/Pc/Fe(CA)	8-2000	2.0	10
Hg-Ag@GNR-PSS-PDDA/GCE (CA)	0.16-1.7	0.12	11
np-Ag (CA)	2500-22500	25.4	12
Porphyrin/SWNTs-[BMIM][PF_6]/GCE (CA)	0.9-140	0.38	13
TH/TNTs/CS/GCE (CV)	15-1500	-	14
$CPE_{Ag/C}$ (cv)	3114 – 5508	326	This work
$CPE_{Ag/C}$ (SWV)	395 – 3114	167	

References

1. Bujes-Garrido J, Izquierdo-Bote D, Heras A, Colina A, Arcos-Martínez MJ. Determination of halides using Ag nanoparticles-modified disposable electrodes. A first approach to a wearable sensor for quantification of chloride ions. Analytica chimica acta. 2018 Jul 5;1012:42-8.

2. Qin X, Wang H, Miao Z, Wang X, Fang Y, Chen Q, Shao X. Synthesis of silver nanowires and their applications in the electrochemical detection of halide. Talanta. 2011 May 15;84(3):673-8.

3. Chiu MH, Cheng WL, Muthuraman G, Hsu CT, Chung HH, Zen JM. A disposable screen-printed silver strip sensor for single drop analysis of halide in biological samples. Biosensors and Bioelectronics. 2009 Jun 15;24(10):3008-13.

4. Choi HG, Laibinis PE. Electrochemical detection of chloride by underpotentially deposited silver films on polycrystalline gold. Analytical chemistry. 2004 Oct 1;76(19):5911-7.

5. Chu L, Zhang X. Electrochemical detection of chloride at the multilayer nano-silver modified indium-tin oxide thin electrodes. Journal of Electroanalytical Chemistry. 2012 Jan 15;665:26-32.

6. Zhang R, Xu S, Zhu Y, Luo J, Liu X, Tang D. One-pot facile preparation of Ag nanoparticles for chloride ion sensing. Colloid and Polymer Science. 2016 Oct;294(10):1643-9.

7. Bashami RM, Soomro MT, Khan AN, Aazam ES, Ismail IM, El-Shahawi MS. A highly conductive thin film composite based on silver nanoparticles and malic acid for selective electrochemical sensing of trichloroacetic acid. Analytica Chimica Acta. 2018 Dec 7;1036:33-48.

8. Liu B, Deng Y, Hu X, Gao Z, Sun C. Electrochemical sensing of trichloroacetic acid based on silver nanoparticles doped chitosan hydrogel film prepared with controllable electrodeposition. Electrochimica acta. 2012 Aug 1;76:410-5.

9. Liu B, Hu X, Deng Y, Yang S, Sun C. Selective determination of trichloroacetic acid using silver nanoparticle coated multi-walled carbon nanotubes. Electrochemistry communications. 2010 Oct 1;12(10):1395-7.

10. Kurd M, Salimi A, Hallaj R. Highly sensitive amperometric sensor for micromolar detection of trichloroacetic acid based on multiwalled carbon nanotubes and Fe (II)–phtalocyanine modified glassy carbon electrode. Materials Science and Engineering: C. 2013 Apr 1;33(3):1720-6.

11. Qian D, Li W, Chen F, Huang Y, Bao N, Gu H, Yu C. Voltammetric sensor for trichloroacetic acid using a glassy carbon electrode modified with Au@ Ag nanorods and hemoglobin. Microchimica Acta. 2017 Jul;184(7):1977-85.

12. Chen T, Liu Z, Lu W, Zhou X, Ma H. Fabrication of free-standing nanoporous silver by selectively dissolving gold from gold–silver alloys via a novel converse dealloying method. Electrochemistry communications. 2011 Oct 1;13(10):1086-9.

13. Tu W, Lei J, Ju H. Functionalization of carbon nanotubes with water-insoluble porphyrin in ionic liquid: direct electrochemistry and highly sensitive amperometric biosensing for trichloroacetic acid. Chemistry–A European Journal. 2009 Jan 5;15(3):779-84.

14. Dai H, Xu H, Wu X, Lin Y, Wei M, Chen G. Electrochemical behavior of thionine at titanate nanotubes-based modified electrode: A sensing platform for the detection of trichloroacetic acid. Talanta. 2010 Jun 15;81(4-5):1461-6.
